# Renal tuberculosis in an imatinib-treated chronic myeloid leukemia

**DOI:** 10.1590/2175-8239-JBN-2019-0123

**Published:** 2020-04-27

**Authors:** Abhilash Chandra, Namrata Rao, Kiran Preet Malhotra

**Affiliations:** 1 Dr.RMLIMS, Department of Nephrology, Vibhuti Khand, Gomti Nagar, Lucknow, 226010, India.; 2 Dr.RMLIMS, Department of Pathology, Vibhuti Khand, Gomti Nagar, Lucknow, 226010, India.

**Keywords:** Imatinib Mesylate, Tuberculosis, Leukemia, Myelogenous, Chronic, BCR-ABL Positive, Mesilato de Imatinib, Tuberculose, Leucemia Mielogênica Crônica BCR-ABL Positiva

## Abstract

Imatinib, which inhibits tyrosine kinase activity of Bcr-Abl protein, is a standard form of treatment for chronic myeloid leukemia (CML). Through its immunomodulatory effect it affects T cell function in a number of ways. It inhibits antigen-induced T cell activation and proliferation. Antigen-specific T-cells and macrophages are vital for protection against *Mycobacterium tuberculosis*. Here we present a case of renal tuberculosis associated with imatinib therapy in the maintenance phase of CML. With granulomatous interstitial nephritis and positive tubercular DNA on renal biopsy, the condition was successfully treated with anti-tubercular therapy. This case provides support to the hypothesis that imatinib therapy in CML increases the susceptibility to tuberculosis and strict vigilance is required to enable its early detection and treatment.

## RESUMO

O imatinibe, um inibidor da atividade da tirosina-quinase da proteína BCR-ABL, faz parte do padrão de tratamento para leucemia mieloide crônica (LMC). Por conta de seu efeito imunomodulador, o imatinibe afeta a função dos linfócitos T de várias maneiras ao inibir a sua ativação e proliferação induzidas por antígenos. Linfócitos T e macrófagos antígeno-específicos são vitais para a proteção contra o *Mycobacterium tuberculosis*. O presente artigo relata um caso de tuberculose renal associada a terapia com imatinibe na fase de manutenção da LMC. Com nefrite intersticial granulomatosa e positividade para DNA de M. tuberculosis na biópsia renal, o paciente foi tratado com sucesso com terapia antituberculínica. O presente caso corrobora a hipótese de que a terapia com imatinibe na LMC aumenta a suscetibilidade à tuberculose, exigindo vigilância rigorosa para permitir sua detecção e tratamento precoces.

## INTRODUCTION

Chronic myeloid leukemia (CML) is a myeloproliferative disorder. In CML, chromosomal translocation of the Bcr gene to Abl gene produces the Bcr-Abl fusion protein. Imatinib, a widely used drug in the treatment of CML, inhibits constitutive tyrosine kinase activity of Bcr-Abl protein.

Tuberculosis (TB) remains one of the biggest health issues in India. The incidence of TB in India is one of the highest in the world: 2.15 million TB cases were notified in India in the year 2018 1. Poor immune status is one of the important risk factors for developing the disease. Other known risk factors include old age, male sex, diabetes mellitus, chronic obstructive airway disease, end stage renal disease 2, chronic liver disease, and certain malignancies. Association of TB with CML is not consistent. Silva et al. reported a very low prevalence of TB in CML patients as compared to other hematological malignancies (2.2%) 3.

Here, we present a case of CML with disseminated TB presenting with features of renal dysfunction and minimal pulmonary symptoms where the used therapeutic drug, imatinib, was a contributing factor.

## CASE

**Figure 1:**
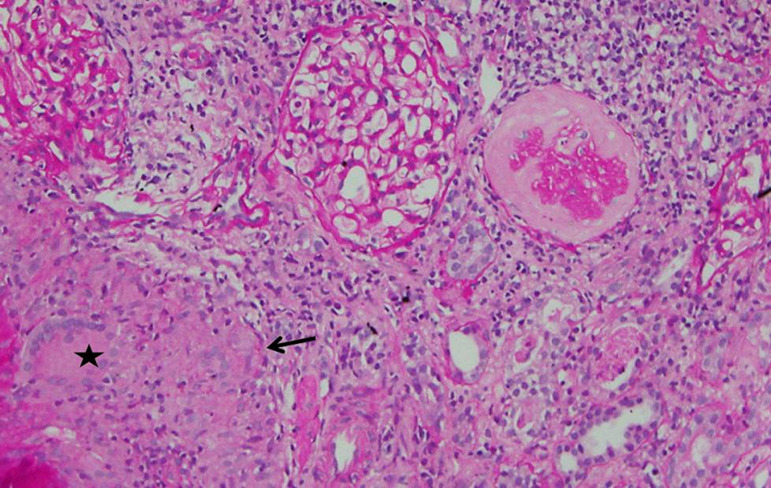
Section from renal biopsy showing two unremarkable and one sclerosed glomerulus. Moderate lymphocytic infiltrate is seen in the interstitium with a granuloma (arrow) and a Langhan's giant cell (asterisk). Periodic Acid Schiff, x200 magnification.

**Figure 2:**
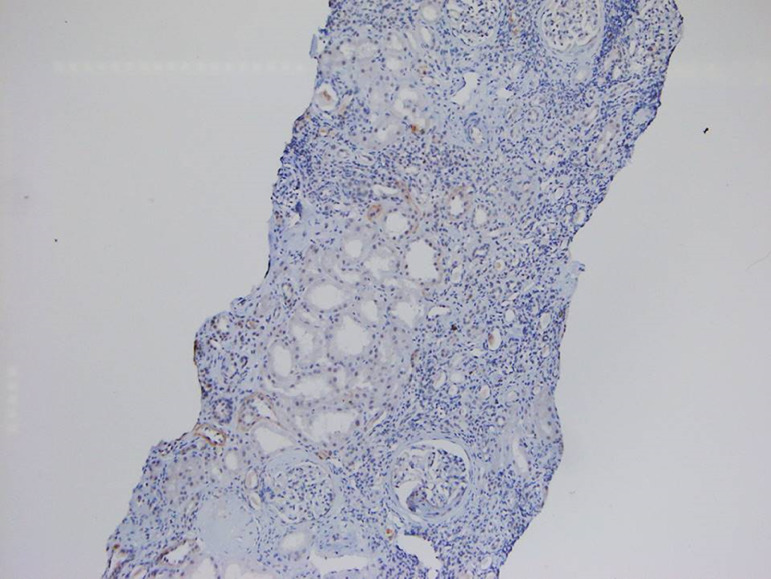
Section from renal biopsy without any CD117-positive cell in the interstitium (diaminobenzidine, x100 magnification).

**Figure 3:**
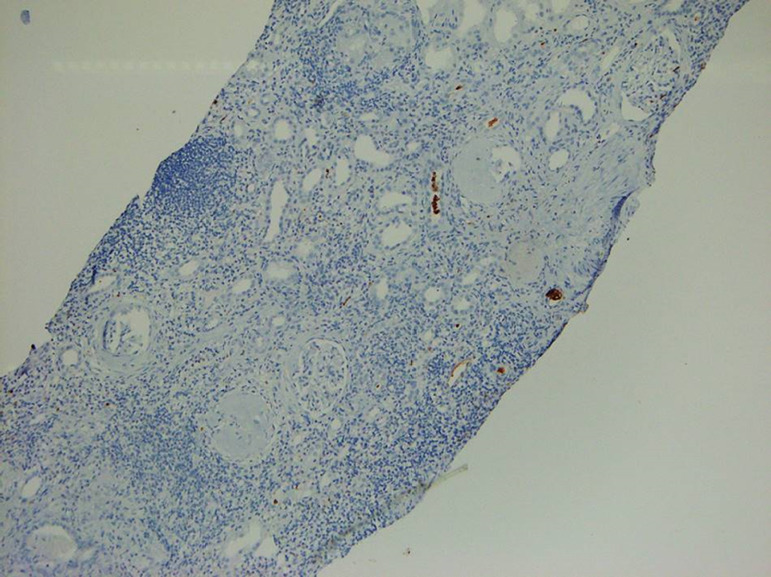
Section from renal biopsy without any myeloperoxidase-positive cell in the interstitium (diaminobenzidine, x100 magnification).

A 29-year-old male was diagnosed as having BCR-ABL-positive CML, and imatinib mesylate therapy was initiated (400 mg/day) about two years ago (age 27 yrs.). Three months post-initiation, the imatinib dosage was increased to 600 mg/day. The patient responded well to the therapy. BCR-ABL/ABL ratio (%) showed a gradual decline from 60.36% at the start of therapy to 0.08% at the time of presentation. During therapy, he developed anorexia and nausea that persisted for 2 months prompting his physician to perform a thorough evaluation. By investigation results he was found to be anemic (Hb-9g/dL). Total leucocyte and platelet counts were normal. Serum creatinine had risen to a value of 1.99 mg/dL from a baseline of 1.1. Serologic test results for human immunodeficiency virus and hepatitis B and C virus were negative, and liver function tests were normal. Urine examination showed 1+ proteinuria with 20-25 rbc/hpf and 4-5 wbc/hpf. With these findings, he was referred to the department of Nephrology. In view of deteriorating renal functions, proteinuria, and active urinary sediments he was taken for a renal biopsy, which revealed granulomatous interstitial nephritis (Figures 1 and 2). No CD117 or myeloperoxidase positive cells were seen in the interstitium (Figures 2 and 3). Finding from Ziehl-Neelsen stain was negative for AFB and Mantoux test was negative. Renal tissue PCR was positive for tubercular DNA. A urine sample was subjected to BACTEC Mycobacteria Growth Indicator Tube [MGIT] culture, which turned out to be positive. A thorax CT scan was done to look for a primary source of TB, which revealed an area of collapse and consolidation in the posterior segment of the right upper lobe and lateral segment of the right middle lobe. The apico-posterior segment of the left upper lobe showed cavitary changes. Multiple enlarged para-aortic, precarinal, and subcarinal lymph nodes were also seen. In spite of the prominent findings in the CT scan, the patient had no symptoms pertaining to his respiratory system. Also, there was no complaint of fever or significant weight loss. He was never before evaluated for latent TB nor had any history of treatment for tuberculosis in the past. Anti-tubercular therapy comprising of isoniazid, rifampicin, pyrazinamide, and ethambutol was initiated. This was followed by symptomatic improvement, and serum creatinine settled to a value of 1.3 mg/dL. Imatinib therapy was continued at a dose of 600 mg/day. Antitubercular therapy was continued for a total of six months.

## DISCUSSION

The relationship between CML and TB is not very clear. While some studies have reported a higher incidence of TB in CML,^4^ others have not found strong evidence of a relationship 3. Possibly, different study designs and regional variations of disease incidence have contributed to the different outcomes.

Being the first line of treatment in CML, imatinib has other immunological implications as well, including an immunomodulatory effect and T cell function modulation in a number of ways. It inhibits antigen-induced T cell activation and proliferation, and inhibits phosphorylation of zap70, a member of the tyrosine kinase family thereby interfering in T cell receptor-related signaling cascade. Cytokine production is also impaired 5. In addition, imatinib has been shown to cause accumulation of cells in G0/G1 phase of the cell cycle and inhibit delayed type hypersensitivity, thereby leading to a rise in number of infections 6. However, these effects have been found to be reversible on discontinuation of the drug 6. Imatinib also impairs the function of antigen-presenting dendritic cells along with recall response of specific memory CD8T cell 7. Reduction of immunoglobulin levels are also seen in patients treated with imatinib. This effect is more pronounced in those with better cytogenetic response, which could be due to B cell dysfunction associated with ABL inhibition by imatinib 8.

Considering the fact that our patient had responded well to imatinib and was on maintenance therapy, the predisposing factor behind his active TB could well have been the drug itself by the above detailed mechanisms.

TB is either acquired through transmission from a person with active disease or activation of a latent infection. The above immune changes brought by imatinib can provide fertile ground for active TB through either route. Although similar cases of TB have been reported with imatinib therapy in CML 9 ^,^ 10 ^,^ to the best of our knowledge renal involvement has never been reported so far. Daniels et al. 9 reported three such cases. Two had pulmonary TB while one presented as a paravertebral mass. The dose of imatinib in these cases varied from 400 to 800 mg/day. Similar to our case, all these responded well to anti-tuberculous therapy (ATT). Another case of meningeal tuberculoma in a patient with CML on imatinib 400 mg/day was reported by Pravin Salunke et al. 11.

The dose required to inhibit T cell proliferation was found to be 400 mg by Dietz et al. 6, which is actually lower than what our patient was taking. This could be one of the reasons behind his susceptibility to TB infection and anergy to Mantoux test. Reduction in the maintenance dosage of imatinib is also a viable option in those who achieve good response in the early phase of the treatment 12 ^,^ 13. This strategy might help negate some of the overt side effects of the drug. As rifampicin is a CYP3A4 inducer, the dose of imatinib needs to be increased by approximately 50% 14. However, in our case imatinib was continued at the same dose of 600 mg/day on account of the low level of CML disease status.

Granulomatous interstitial nephritis has been found to be associated with antibiotics, analgesics, and infections like tuberculosis, fungal infections, sarcoidosis, and granulomatosis with polyangiitis 15. In our case, positivity of tissue PCR for tubercular DNA, positive urine culture for *Mycobacterium tuberculosis* and good response to therapy clearly indicated the tubercular cause of granulomas in the renal tissue. Absence of CD117 and myeloperoxidase-positive cells in the interstitium rules out the possibility of myelogenous cause of the reported interstitial nephritis. Similar findings in cases of renal tuberculosis have also been reported by Daher et al. 16.

## CONCLUSION

This case raises the hypothesis that imatinib therapy in CML can increase the susceptibility to infections including TB, probably by affecting the acquired immunity through its influence on specific T cells. Assessment of the risk of TB infection prior to imatinib therapy can help in preventing the disease. Atypical clinical presentation of TB infection in such cases necessitates high degree of suspicion, particularly in endemic regions.

## References

[1] Ministry of Health and Family Welfare (IND) (2018). TB India Report 2018: Central of Tuberculosis Division - Government of India.

[2] Hu HY, Wu CY, Huang N, Chou YJ, Chang YC, Chu D (2014). Increased risk of tuberculosis in patients with end-stage renal disease: a population-based cohort study in Taiwan, a country of high incidence of end-stage renal disease. Epidemiol Infect.

[3] Silva FA, Matos JO, Mello FCQ, Nucci M (2005). Risk factors for and attributable mortality from tuberculosis in patients with hematologic malignances. Haematologica.

[4] Liu CJ, Hong YC, Teng CJ, Hung MH, Hu YW, Ku FC (2015). Risk and impact of tuberculosis in patients with chronic myeloid leukemia: a nationwide population-based study in Taiwan. Int J Cancer.

[5] Seggewiss R, Loré K, Greiner E, Magnusson MK, Price DA, Douek DC (2005). Imatinib inhibits T-cell receptor-mediated T-cell proliferation and activation in a dose-dependent manner. Blood.

[6] Dietz AB, Souan L, Knutson GJ, Bulur PA, Litzow MR, Vuk-Pavlovic S (2004). Imatinib mesylate inhibits T-cell proliferation in vitro and delayed-type hypersensitivity in vivo. Blood.

[7] Sinai P, Berg RE, Haynie JM, Egorin MJ, Ilaria RL, Forman J (2007). Imatinib mesylate inhibits antigen-specific memory CD8 T cell responses in vivo. J Immunol.

[8] Steegmann JL, Moreno G, Aláez C, Osorio S, Granda A, Cámara R (2003). Chronic myeloid leukemia patients resistant to or intolerant of interferon alpha and subsequently treated with imatinib show reduced immunoglobulin levels and hypogammaglobulinemia. Haematologica.

[9] Daniels JM, Vonk-Noordegraaf A, Janssen JJ, Postmus PE, Van Altena R (2009). Tuberculosis complicating imatinib treatment for chronic myeloid leukaemia. Eur Respir J.

[10] Ghadyalpati N, Prabhash K, Menon H, Nair R, Banavali S, Noronha V (2010). Tuberculosis infection in chronic myeloid leukemia (CML) patients treated with imatinib. J Clin Oncol.

[11] Salunke P, Gupta K, Singla N, Singh H, Singh P, Mukherjee KK (2011). Meningeal tuberculoma mimicking chloroma in a patient with chronic myeloid leukemia on imatinib.. Neurol India.

[12] Petzer AL, Fong D, Lion T, Dyagil I, Masliak Z, Bogdanovic A (2012). High-dose imatinib induction followed by standard-dose maintenance in pre-treated chronic phase chronic myeloid leukemia patients - final analysis of a randomized, multicenter, phase III trial. Haematologica.

[13] Faber E, Divoká M, Skoumalová I, Novák M, Marešová I, Mičová K (2016). A lower dosage of imatinib is sufficient to maintain undetectable disease in patients with chronic myeloid leukemia with long-term low-grade toxicity of the treatment. Leuk Lymphoma.

[14] Sorà F, Matteis S, Di Mario A, Maiuro G, Laurenti L, Chiusolo P (2006). Antituberculosis therapy and imatinib for chronic myeloid leukemia. Clin Infect Dis.

[15] Shan S, Carter-Monroe N, Atta MG (2015). Granulomatous interstitial nephritis. Clin Kidney J.

[16] Ede FD, Silva GB, Barros EJ (2013). Renal tuberculosis in the modern era. Am J Trop Med Hyg.

